# *In Vitro* Evaluation of the Potential Interactions of Zearalenone-14-sulfate and Zearalenone-14-glucuronide with Human Cytochrome P450 Enzymes, Organic Anion Transporting Polypeptides, and ATP-Binding Cassette Multidrug Transporters

**DOI:** 10.1021/acsomega.5c05217

**Published:** 2025-07-16

**Authors:** Ágnes Telbisz, Hana Kaci, Éva Bakos, Zoltán Nagymihály, Eszter B. Both, Nándor Lambert, Csilla Özvegy-Laczka, Miklós Poór

**Affiliations:** † Gene Regulation Research Group, Institute of Molecular Life Sciences, 280964 Research Centre for Natural Sciences, HUN-REN, H-1117 Budapest, Hungary; ‡ Drug Resistance Research Group, Institute of Molecular Life Sciences, Research Centre for Natural Sciences, HUN-REN, H-1117 Budapest, Hungary; § Doctoral School of Biology, Institute of Biology, Eötvös Loránd University, H-1117 Budapest, Hungary; ∥ 61535 Soft Flow Ltd., Pellérdi út 91/B, H-7634 Pécs, Hungary; ⊥ Molecular Medicine Research Group, János Szentágothai Research Centre, 37656 University of Pécs, Ifjúság útja 20, H-7624 Pécs, Hungary; # National Laboratory on Human Reproduction, University of Pécs, Ifjúság útja 20, H-7624 Pécs, Hungary; ∇ Department of Laboratory Medicine, Medical School, University of Pécs, Ifjúság útja 13, H-7624 Pécs, Hungary

## Abstract

Zearalenone (ZEN) is a mycotoxin that is typically produced by *Fusarium* strains. ZEN and its derivatives are common food contaminants and known xenoestrogens. Previous studies demonstrated the interactions of ZEN and zearalenols with certain cytochrome P450 (CYP) enzymes, organic anion transporting polypeptides (OATPs), and ATP-binding cassette (ABC) multidrug transporters. However, no data are available regarding the conjugated metabolites of the mycotoxin. Therefore, in the current study, we aimed to investigate the potential interactions of zearalenone-14-sulfate (Z14S) and zearalenone-14-glucuronide (Z14GA) with these proteins using *in vitro* assays. Our major observations/conclusions are the following: Z14S was a weak inhibitor of CYP2C9 and CYP3A4, while Z14GA did not affect the activity of the CYP enzymes examined. Z14S inhibited OATP1A2 and OATP1B3 at low micromolar concentrations, and it showed even stronger effects on OATP1B1 and OATP2B1 with nanomolar IC_50_ values. In contrast, Z14GA proved to be a weak inhibitor of OATPs tested, except OATP2B1. Among the ABC transporters investigated, we noticed the most relevant interactions of ZEN with MDR1, Z14S with BCRP, and Z14GA with MRP2. Our novel observations may contribute to the deeper understanding of the toxicokinetic interactions of Z14S and Z14GA.

## Graphical abstract

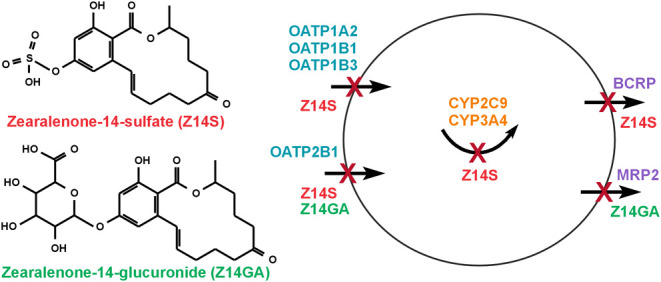

## 1 Introduction

Zearalenone (ZEN; Figure 1) is a mycotoxin produced by *Fusarium* fungi. ZEN (and some of its derivatives) is a common food contaminant that usually occurs in cereal grains and the corresponding food products.^1–3^ ZEN is a nonsteroid macrolide, it can activate estrogen receptors, and other mechanisms may also be involved in its endocrine disruptor effects.^1,2,4–6^ In addition, certain studies suggest that ZEN may have hepatotoxic, immunotoxic, and genotoxic impacts.^2^ Both phase I and phase II reactions take part in the biotransformation of ZEN. The most dominant phase I metabolites are produced by 3α- or 3β-hydroxysteroid dehydrogenases; these reduced derivatives are α-zearalenol (α-ZEL), β-zearalenol (β-ZEL), zearalanone (ZAN), α-zearalanol (α-ZAL), and β-zearalanol (β-ZAL).^1^ Importantly, α derivatives (α-ZEL and α-ZAL) have much higher xenoestrogenic potency compared to the parent mycotoxin.^1,2^ Cytochrome P450 (CYP) enzymes have lower involvement in the biotransformation of ZEN; however, during CYP-catalyzed reactions, catechol and then quinone derivatives are formed.^1,7^ These metabolites are weaker xenoestrogens than ZEN, but the DNA-reactive quinones may have high importance in ZEN-induced genotoxicity.^7,8^ The most relevant phase II metabolites of ZEN are its glucose, sulfate, and glucuronide conjugates.^1^ Zearalenone-14-glucoside and zearalenone-14-sulfate (Z14S; Figure 1) are masked/modified derivatives of the mycotoxin produced by plant and fungal metabolism, respectively.^1,9^ Zearalenone-14-glucuronide (Z14GA; Figure 1) and other glucuronic acid metabolites of reduced ZEN derivatives are formed in mammals by uridine 5′-diphospho-glucuronosyltransferases (UGTs); these derivatives are typically subjected to biliary excretion and enterohepatic cycling.^1^ Furthermore, in mammals, Z14S (and other sulfate metabolites) can also be produced by sulfotransferases (SULTs).^1,10,11^ The conjugated metabolites are weaker xenoestrogens than the parent mycotoxin; however, in the gastrointestinal tract, their close to complete hydrolysis to ZEN has been reported.^1,9^

**Figure 1:**
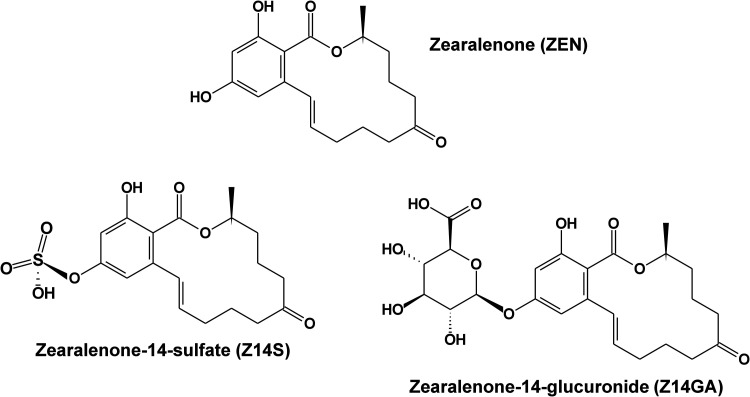
Chemical structures of zearalenone, zearalenone-14-sulfate, and zearalenone-14-glucuronide.

CYP enzymes take part in the biotransformation of more than 70% of medications and they are also frequently involved in the metabolism of other xenobiotics.^12,13^ Previous studies demonstrated the interactions of ZEN and ZELs with certain CYP enzymes. ZEN, α-ZEL, and β-ZEL showed moderate inhibitory effects on CYP1A2 and CYP2C9 (IC_50_ ≈ 6–20 μM), and their low micromolar IC_50_ values (1.4–2.0 μM) were determined regarding CYP3A4.^14^ The few studies available suggest that CYP1A2 and/or CYP3A4 are the most important CYP enzymes in the biotransformation of ZEN.^8,14,15^ Interestingly, in mycotoxin depletion assays, ZEN and β-ZEL concentrations were decreased in the presence of both CYP1A2 and CYP3A4 enzymes, while α-ZEL levels were only reduced by CYP1A2.^14^

Organic anion transporting polypeptides (OATPs) are drug transporters, and they have an important role in the absorption, tissue uptake, and/or excretion of numerous compounds, including steroid hormones, medications, nutrients, and toxins.^16,17^ OATP1B1 and OATP1B3 are expressed on the basolateral membrane of hepatocytes and mediate the hepatic clearance of many substrates,^18,19^ while OATP1A2 and OATP2B1 take part in the intestinal absorption and blood–brain barrier penetration.^19–21^ Based on an earlier report, ZEN and ZELs can inhibit OATP1A2, OATP1B1, OATP1B3, and OATP2B1 transporters, where these mycotoxins caused marked decreases in the transport activity of OATP1A2 and OATP2B1 even at low micromolar concentrations (IC_50_ = 1.5–3.2 μM).^14^ Nevertheless, ZEN and ZELs did not show higher toxicity in OATP-overexpressing A431 cells compared to the mock controls, suggesting no relevant involvement of OATP1A2, OATP1B1, OATP1B3, or OATP2B1 in the cellular uptake of these mycotoxins.^14^

ATP-binding cassette (ABC) multidrug transporters P-glycoprotein (MDR1/P-gp/ABCB1), breast cancer resistance protein (BCRP/ABCG2), and multidrug resistance-associated protein 2 (MRP2/ABCC2) are located in tissue barriers, e.g., canalicular surfaces of hepatocytes, kidneys, and the placenta. These ABC transporters are involved in the excretion of several toxic compounds, including their sulfate and glucuronide metabolites.^22–26^ Drug exclusion at placental and blood–brain barriers is primarily driven by MDR1 and BCRP exporters.^27^ MDR1 is a main transporter of structurally unrelated hydrophobic compounds and has a partially overlapping specificity with that of BCRP. BCRP can handle organic anions and conjugates besides hydrophobic substrates.^27^ Furthermore, MRP2 is a transporter of organic anions, among them drug conjugates.^23^

Intestinal uptake and placental crossing of mycotoxins are important issues, where MDR1, BCRP, and MRPs can modify absorption and tissue distribution. MDR1 and MRPs were investigated in relevant model cell lines, such as Caco-2 cells or transporter overexpressing lines of LLCPK1 and MDCKII.^28^ It was found that MDR1 has no significant role in ZEN and ZEL transport, but MRPs are able to transport these compounds. Differential investigations of MRPs in models revealed that ZEN and α-ZEL are transported by MRP1 (ABCC1), ZEN and α-/β-ZELs by MRP2 (ABCC2), while only β-ZEL by MRP3 (ABCC3).^28^

In another study, the BCRP-mediated transport of ZEN was investigated in various experimental models.^29^ BCRP-related transport was studied in BeWo placental cells with intrinsic BCRP expression and in transporter-specific vesicular transport assays, and both results concluded that ZEN is an inhibitor of BCRP-mediated transport of labeled substrates. Furthermore, in the HEK cell model, the cells with the Q141 K SNP variant were more sensitive to ZEN toxicity compared to the cells expressing the wild-type ABCG2 gene.^29^ Q141 K is a human SNP variant that is quite common in clinical samples, and it causes the reduced expression of the BCRP protein.^30^ A following study demonstrated both human and mouse BCRP/Bcrp-mediated transepithelial ZEN transport in overexpressing MDCK cells and in BCRP expressing BeWo choriocarcinoma cells.^31^ In BeWo cells, the transepithelial transport of ZEN was inhibited by BCRP shRNA. In addition, in pregnant mice, higher ZEN, α-ZEL, and β-ZEL levels were found in the placenta of Bcrp–/– mice and in their fetuses compared to the wild-type animals.^31^

The toxicokinetic interactions of ZEN and ZELs with CYP enzymes and with OATP and ABC transporters have been examined in few earlier studies. However, despite the fact that glucuronide conjugation is the dominant phase II metabolic pathway of ZEN and its reduced derivatives in humans and animals,^1,32^ we did not find data regarding the interactions of ZEN conjugates with these proteins. Therefore, to get a deeper insight, the impacts of Z14S and Z14GA on CYP (CYP1A2, CYP2C9, and CYP3A4) enzymes, on OATPs (OATP1A2, OATP1B1, OATP1B3, and OATP2B1), and on ABC (MDR1, BCRP, and MRP2) transporters were examined using *in vitro* models.

## 2 Materials and Methods

### 2.1 Reagents

Zearalenone-14-sulfate (Z14S) was purchased from ASCA GmbH (Berlin, Germany). Zearalenone-14-glucuronide (Z14GA) was synthesized as it has been reported.^33^ Zearalenone (ZEN), CypExpress Cytochrome P450 (CYP2C9 and CYP3A4) human kits, α-naphthoflavone, testosterone, 6β-hydroxytestosterone, ketoconazole, nicotinamide adenine dinucleotide phosphate (NADP^+^), glucose-6-phosphate (G6P), pyranine, sulforhodamine 101 (SR101), *N*-methylquinidine (NMQ), lucifer yellow (LY), 5(6)-carboxy-2′,7′-dichlorofluorescein (CDCF), verapamil, benzbromarone, and further chemicals, if not stated otherwise, were obtained from Merck (Darmstadt, Germany). Diclofenac, 4′-hydroxydiclofenac, and sulfaphenazole were from Biosynth (Berkshire, U.K.). Dulbecco’s Modified Eagle Medium (DMEM) was purchased from Thermofisher Scientific (Waltham, MA). Ko143 was obtained from Tocris Bioscience (Bristol, U.K.).

### 2.2 CYP Assays

We applied 10 mM stock solutions of mycotoxins (dissolved in DMSO), and the samples (including controls and positive controls) contained uniformly 0.2 vol % DMSO. In CYP assays, α-naphthoflavone (CYP1A2), sulfaphenazole (CYP2C9), and ketoconazole (CYP3A4) were used as positive control inhibitors. Effects of ZEN, Z14S, and Z14GA on CYP1A2 activity were tested applying the Fluorometric CYP1A2 Inhibitor Assay Kit (ab211075; Abcam, Cambridge, U.K.),^14,34^ following exactly the manufacturer’s instructions.

Inhibitory actions of ZEN conjugates on CYP2C9 and CYP3A4 enzymes were examined employing CypExpress Cytochrome P450 human kits.^14,34^ Briefly, incubates (200 μL) contained the substrate (5 μM; diclofenac for CYP2C9 and testosterone for CYP3A4), the CypExpress reagent (6 mg/mL of CYP2C9 kit or 15 mg/mL of CYP3A4 kit; also including the glucose-6-phosphate dehydrogenase enzyme), and the test compounds (each 20 μM) in 0.05 M potassium phosphate buffer (pH 7.5). CYP3A4 assay was supplemented with additional G6P and NADP^+^ (both 400 μM). Enzyme assays were started with the addition of the enzyme. After 120 min (CYP2C9) or 180 min (CYP3A4) incubation at 700 rpm and 30 °C in a thermomixer, the reaction was stopped with 100 μL of ice-cold methanol. Then, the samples were centrifuged for 10 min at 14,000*g* and room temperature. The supernatants were gently removed and directly analyzed by HPLC (see Section 2.3).

### 2.3 HPLC-UV Analyses

HPLC analyses were executed employing an integrated HPLC system (Jasco, Tokyo, Japan) built up from an autosampler (AS-4050), a binary pump (PU-4180), a UV detector (UV-975), and ChromNAV2 software.

Diclofenac and 4′-hydroxydiclofenac (CYP2C9 assay) were quantified using the previously reported HPLC-UV assay,^14,34^ without modification. Briefly, the isocratic elution, with 1.0 mL/min flow rate, was performed with orthophosphoric acid (6 mM) and acetonitrile (48:52 v/v %) mobile phase, applying a Security Guard (C8, 4.0 × 3.0 mm; Phenomenex, Torrance, CA) precolumn and a Mediterranea Sea8 (C8, 150 × 4.6 mm, 5 μm; Teknokroma, Barcelona, Spain) analytical column at room temperature (injected volume: 20 μL). Diclofenac and 4′-hydroxydiclofenac were detected at 275 nm.

Testosterone and 6β-hydroxytestosterone (CYP3A4 assay) were quantified using the previously reported HPLC assay,^14,34^ without modification. Briefly, the isocratic elution, with 1.2 mL/min flow rate, was performed with methanol, water, and acetic acid (53:46:1 v/v%) mobile phase, applying a Security Guard (C18, 4.0 × 3.0 mm; Phenomenex) precolumn and a Kinetex EVO C18 (150 × 4.6 mm, 5 μm; Phenomenex) analytical column at room temperature (injected volume: 20 μL). Testosterone and 6β-hydroxytestosterone were detected at 240 nm.

### 2.4 Effects of Z14S and Z14GA on the Activities of OATP1A2, OATP1B1, OATP1B3, and OATP2B1

The human epidermoid carcinoma cell (A431) overexpressing OATP1A2, OATP1B1, OATP1B3, or OATP2B1 transporters and their mock control cells were generated previously as described.^35,36^ Cell culture was maintained in DMEM supplemented with 10% fetal bovine serum, 2 mM l-glutamine, 100 units/mL penicillin, and 100 μg/mL streptomycin at 37 °C and 5% CO_2_.

The inhibitory effects of mycotoxins (Z14S and Z14GA) were examined using indirect fluorescence assays, where pyranine and SR101 were used as fluorescent substrates.^36,37^ A431 cells overexpressing OATP1A2, OATP1B1/3, or OATP2B1 and their mock-transfected controls were seeded at a density of 8 × 10^4^ cells/well onto 96-well plates and incubated for 18–22 h prior to transport measurements. The next day, plated cells were washed three times with 200 μL of PBS (phosphate-buffered saline, pH 7.4) and preincubated with 50 μL of transport buffer (pH 5.5; 125 mM NaCl, 4.8 mM KCl, 1.2 mM CaCl_2_, 1.2 mM KH_2_PO_4_, 12 mM MgSO_4_, 25 mM MES, and 5.6 mM glucose) with or without increasing concentrations of the mycotoxins tested (0.001–20 μM) for 5 min at 37 °C. The transport inhibition measurements were initiated by adding 50 μL of transport buffer containing the proper fluorescent substrate SR101 (0.5 μM, OATP1A2) or pyranine (10 μM for OATP1B1 or 20 μM for OATP1B3 and OATP2B1). After 10 min (OATP1A2), 15 min (OATP1B1 and OATP2B1), or 30 min (OATP1B3) incubation, the transport was terminated by washing the cells three times with ice-cold PBS. Regarding the individual OATP assays, we applied different incubation times to keep the substrate uptake in the linear range,^35,36^ avoiding substrate depletion or saturation effects, as it is indispensable for the accurate assessment. Using an Enspire plate reader (PerkinElmer, Waltham, MA), the fluorescence was measured at excitation/emission wavelengths of 460/510 nm (pyranine) or 586/605 nm (SR101). The uptake of the test substrates by OATP-expressing vs the corresponding mock control cells is demonstrated in Figure S1.

OATP-mediated transport was obtained by subtracting the activity of the mock control cells from the activity of the OATP-expressing cells. The transport activity (%) of the OATPs was calculated by comparing the fluorescence signal obtained in the absence of the compounds tested (set to 100%).

### 2.5 Transport Activity Measurements for ABC Transporters

Experiments were performed in insect membrane vesicles, as described previously.^38^ Briefly, human MDR1, BCRP, and MRP2 were expressed in Sf9 insect cells by baculoviruses. On the third day of infection, cells were collected and membrane vesicles were obtained by mechanical disruption and differential centrifugation. To get full activity for BCRP, the cholesterol levels of the vesicles were adjusted to the level of mammalian membranes.^38^ Vesicles were stored at −80 °C, and the total protein content of the preparations was measured by the Lowry method (used as a reference for the quantity). Membrane vesicles (50 μg protein/sample) were incubated at 37 °C for 10 min (without or with 4 mM Mg-ATP) in 50 μL volume in the presence of transporter-specific fluorescent substrates: *N*-methylquinidine (NMQ; 5 μM) for MDR1, lucifer yellow (LY; 10 μM) for BCRP, and 5(6)-carboxy-2′,7′-dichlorofluorescein (CDCF; 5 μM) for MRP2. The quality of membrane vesicles was confirmed by applying known reference inhibitors (verapamil for MDR1, Ko143 for BCRP, and benzbromarone for MRP2). ATP-dependent (4 mM) uptake of fluorescent substrates was examined in the presence of 0–50 μM concentrations of mycotoxins (ZEN, Z14S, or Z14GA). Each compound was dissolved in DMSO and solvent controls were applied in all experiments; the final concentrations of DMSO did not exceed 2 vol %, which did not affect the measurements. After incubation, samples were rapidly filtered and washed on a filter plate (MSFBN6B10; Millipore, Burlington, MA). Accumulated substrates in vesicles were solved back from the filter with 100 μL of SDS (10%) and centrifuged into another plate. A 100 μL volume of fluorescence stabilizer was added to the samples (DMSO for LY, 0.1 M H_2_SO_4_ for NMQ, and 0.1 M NaOH for CDCF). Fluorescence of samples was measured by plate readers (Victor X3 and Enspire PerkinElmer; Waltham, MA) at appropriate wavelengths (filters in the Victor X3 reader were 405/535 nm for LY and 492/635 nm for CDCF; while in the Enspire reader, NMQ was measured at 360/430 nm excitation/emission wavelengths). ABC-related transport was calculated by subtracting passive uptake measured without Mg-ATP from values measured in the presence of Mg-ATP.

### 2.6 ATPase Activity Assays for MDR1 and BCRP Transporter Interaction

ATPase activity was determined in Sf9 membrane vesicles containing human MDR1 or human BCRP prepared as described.^38–42^ Appropriate amounts of vesicles (10 μg/50 μL) were used in the assays. Membrane vesicles were incubated with Mg-ATP (3 mM) for 25 min at 37 °C. Effects of the test compounds were examined up to 50 μM concentrations. Solvent controls were applied in each experiment (DMSO concentration was uniformly 2% v/v, which did not modify the basal activity). ABC transporter function was determined as vanadate-sensitive ATPase activity. Liberated inorganic phosphate (Pi) was measured by a colorimetric reaction, as it has been reported.^39^ After 25 min, absorbance values were determined at 660 nm by a VictorX plate reader (PerkinElmer).

### 2.7 Data Analyses

Mean and standard deviation (±SD) values are derived from at least three independent experiments. Statistical analyses were performed based on one-way ANOVA with Tukey’s post hoc test, applying the SPSS Statistics software (IBM, Armonk, NY). IC_50_ values were determined with sigmoidal fitting (Hill1) using the Origin 8.5 software (OriginLab Corporation, Northampton, MA).

## 3 Results and Discussion

### 3.1 Inhibitory Effects of Z14S and Z14GA on CYP1A2, CYP2C9, and CYP3A4 Enzymes

In our previous study, ZEN exerted moderate to relatively strong inhibitory actions on CYP1A2 (IC_50_ = 7.8 μM), CYP2C9 (IC_50_ = 9.8 μM), and CYP3A4 (IC_50_ = 2.0 μM).^14^ Therefore, we examined the impacts of Z14S and Z14GA on these CYP enzymes. To get a first insight, high levels (each 20 μM) of ZEN, Z14S, Z14GA, and known inhibitors were tested. Under these conditions, the positive control inhibitors (α-naphthoflavone for CYP1A2, sulfaphenazole for CYP2C9, and ketoconazole for CYP3A4) caused more than 90% inhibition in each assay (Figure 2). Z14GA had no effects on the CYP-catalyzed reactions examined, and Z14S did not influence CYP1A2 activity (Figure 2A). However, the statistically significant inhibitory actions of Z14S were observed regarding CYP2C9 and CYP3A4, causing an approximately 45% decrease in metabolite formation in both assays (Figure 2B,C). Since the inhibitory effects of Z14S were less than 50% at 20 μM concentrations, we consider Z14S a weak inhibitor of CYP2C9 and CYP3A4. Therefore, we did not perform further concentration-dependent measurements.

**Figure 2:**
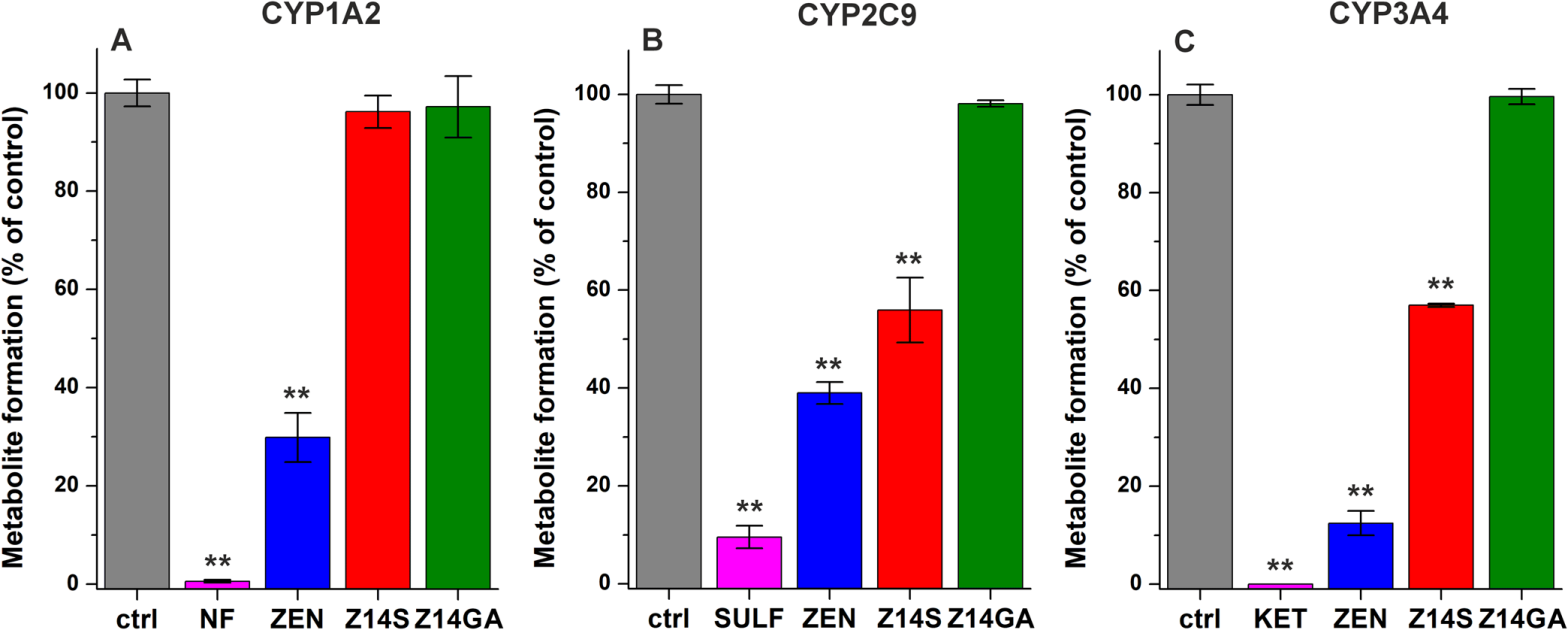
Probing the interactions of ZEN, Z14S, and Z14GA with CYP1A2 (A), CYP2C9 (B), and CYP3A4 (C) enzymes. Effects of positive control inhibitors (magenta; α-NF, α-naphthoflavone; SULF, sulfaphenazole; KET, ketoconazole), ZEN (blue), Z14S (red), and Z14GA (green) (each 20 μM). Data represent means ± SD (*n* = 3; **p < 0.01), all samples contained uniformly 0.2 v/v% DMSO.

Glucuronidation completely abolished the inhibitory effects of ZEN on CYP1A2, CYP2C9, and CYP3A4. It can be explained by the bulky hydrophilic structure of glucuronic acid, which likely prevents the interaction of Z14GA with these proteins. Sulfate conjugation also ceased the inhibitory effect of ZEN on CYP1A2. However, high levels of Z14S were able to inhibit the activities of CYP2C9 and CYP3A4, even if the sulfate derivative proved to be a less potent inhibitor, compared to the parent mycotoxin. Unfortunately, we did not find data regarding the typical plasma concentrations of Z14S; however, earlier studies suggest that the glucuronide metabolites of ZEN and ZELs are the dominant conjugates that appear in human blood and urine.^1^ Therefore, it is unlikely that Z14S could affect the CYP2C9- or CYP3A4-catalyzed biotransformation of drugs or other xenobiotics.

### 3.2 Inhibitory Effects of Z14S and Z14GA on OATP1A2, OATP1B1, OATP1B3, and OATP2B1

To assess the potential inhibitory effects of Z14S and Z14GA on OATPs, we examined the influence of these mycotoxins on OATP-mediated dye uptake using A431 cells overexpressing OATP1A2, OATP1B1, OATP1B3, or OATP2B1. At a 20 μM concentration, each OATP was considerably inhibited by Z14S, resulting in 80–100% decreases in their transport activity (Figure 3). In addition, Z14S completely abolished the OATP2B1-mediated dye uptake at a 5 μM concentration (Figure 3D). Z14S showed low micromolar IC_50_ values for OATP1A2 (IC_50_ = 1.4 μM) and OATP1B3 (IC_50_ = 2.3 μM). Moreover, Z14S proved to be a potent inhibitor of OATP1B1 (IC_50_ = 0.27 μM) and OATP2B1 (IC_50_ = 0.34 μM), markedly reducing the rate of the OATP-mediated dye uptake even at nanomolar levels. Z14GA exerted only weak inhibitory effects on OATP1A2, OATP1B1, and OATP1B3 (IC_50_ > 20 μM); however, it was a relatively strong inhibitor of OATP2B1 (IC_50_ = 1.1 μM) (Figure 3).

**Figure 3:**
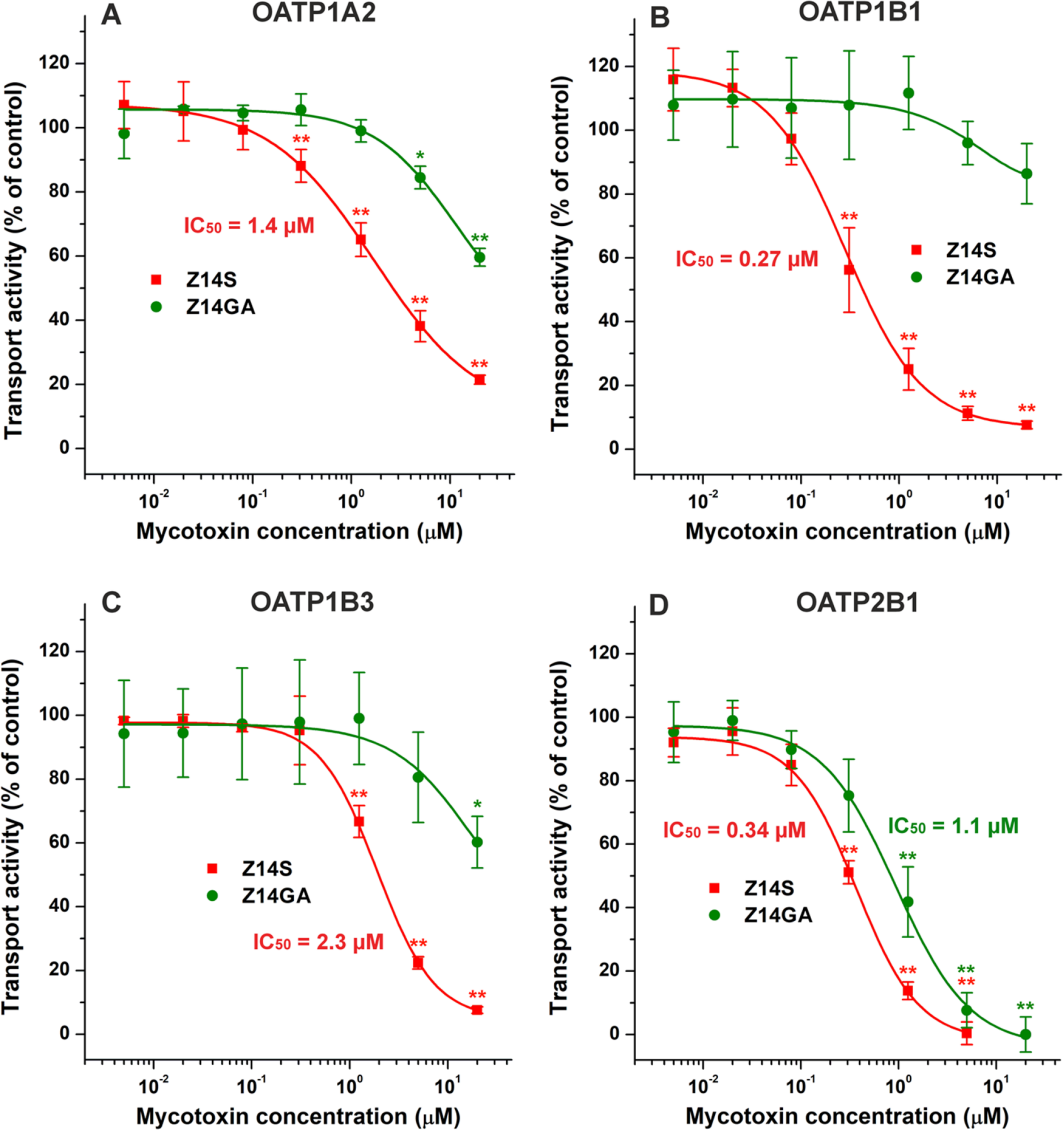
Probing the interactions of Z14S and Z14GA with OATP1A2 (A), OATP1B1 (B), OATP1B3 (C), and OATP2B1 (D). Effects of Z14S (red) and Z14GA (green) on the transport activity of OATPs in overexpressing A431 cells, applying SR101 (OATP1A2) or pyranine (OATP1B1/3 and OATP2B1) as test substrates. Mean transport activity data (% of control ± SD) are represented, where the fluorescence with the dyes alone (without mycotoxins) was set as 100% (*n* = 3; *p < 0.05, **p < 0.01).

In the same experimental model applied in the current study, ZEN was a relatively strong inhibitor of OATP1A2 (IC_50_ = 2.3 μM) and OATP2B1 (IC_50_ = 2.3 μM), and a moderate inhibitor of OATP1B1 (IC_50_ > 10 μM) and OATP1B3 (IC_50_ = 8.1 μM).^14^ These data demonstrate that Z14GA is a weaker inhibitor of OATP1A2, OATP1B1, and OATP1B3 than ZEN. However, regarding OATP2B1, the inhibitory potency of the glucuronide conjugate was slightly higher compared to the parent mycotoxin. Sulfate conjugation resulted in stronger inhibitory effects on each OATP tested (Figure 2).^14^ Importantly, compared to ZEN, the sulfate conjugate exerted approximately 7-fold and more than 30-fold stronger inhibition on OATP2B1 and OATP1B1, respectively. These results are in accordance with our earlier observations with natural polyphenols, where sulfate conjugation typically led to stronger (or at least similar) inhibitory effects on OATPs, while glucuronide metabolites can be weaker or sometimes even stronger inhibitors of these transporters compared to the parent aglycones.^43–45^

Z14S and Z14GA appear in the intestinal tract due to peroral exposure or through the biliary excretion of the conjugate.^1^ Considering the involvement of OATP1A2 and OATP2B1 in the intestinal absorption of certain molecules^19,20^ as well as the inhibitory actions of Z14S and/or Z14GA observed on these transporters (Figure 3), the OATP-mediated active uptake of some compounds may be affected by ZEN conjugates.

After the treatment of samples with glucuronidase, the total plasma levels of ZEN and ZELs were from the nanomolar to the low micromolar concentrations in humans,^46^ demonstrating that glucuronide metabolites can achieve even micromolar levels in the circulation. Z14GA was a weak inhibitor of OATP1B1 and OATP1B3 hepatic transporters; while it showed relatively strong inhibitory effects on OATP2B1, which (transporter) is involved in hepatic uptake and blood–brain barrier penetration of certain xenobiotics.^19,21^ Based on these data, Z14GA may influence these OATP2B1-mediated processes. We did not find data regarding the plasma levels of Z14S in humans or animals. If higher levels of Z14S are produced in certain species, then this conjugate may affect the tissue uptake of other compounds, mainly through the inhibition of OATP1B1- and/or OATP2B1-mediated transport.

### 3.3 Interaction of Z14S and Z14GA with Human ABC (MDR1, BCRP, and MRP2) Transporters

Interaction of ZEN and its metabolites, Z14S and Z14GA, with human ABC drug transporters was investigated *in vitro* in cell membrane-based assays. Human ABC transporters (MDR1, BCRP, or MRP2) were expressed in insect cells by the baculovirus expression method, and cell membrane vesicles were prepared from cells. ABC proteins in inverted cell membrane vesicles can take up their specific fluorescent substrates into the vesicles in an ATP-driven process. Interactions of mycotoxins with MDR1, BCRP, and MRP2 were examined quantitatively by inhibition of fluorescent substrate uptake.

The MDR1-mediated, ATP-dependent uptake of NMQ was inhibited by 97% in the presence of 30 μM verapamil (a known reference substrate). As it is demonstrated in Figure 4A, the MDR1-driven transport is barely influenced by ZEN and its conjugates, and ZEN caused inhibition only at 50 μM concentration. Importantly, NMQ is the sole available fluorescent substrate of MDR1 for the membrane vesicular assay (other, more hydrophobic fluorescent substrates have too high passive permeability), but the sensitivity of the assay is supposed to be low. For better characterization of the potential interactions of ZEN and/or its conjugates with MDR1, we also performed an ATPase assay, applying progesterone as a reference substrate. In agreement with the results of the NMQ transport assay, only ZEN stimulated the drug transport-coupled ATPase activity (Figure 4B), showing considerable elevation even at 5 μM concentration. Z14S and Z14GA did not modify the ATPase activity of MDR1.

**Figure 4:**
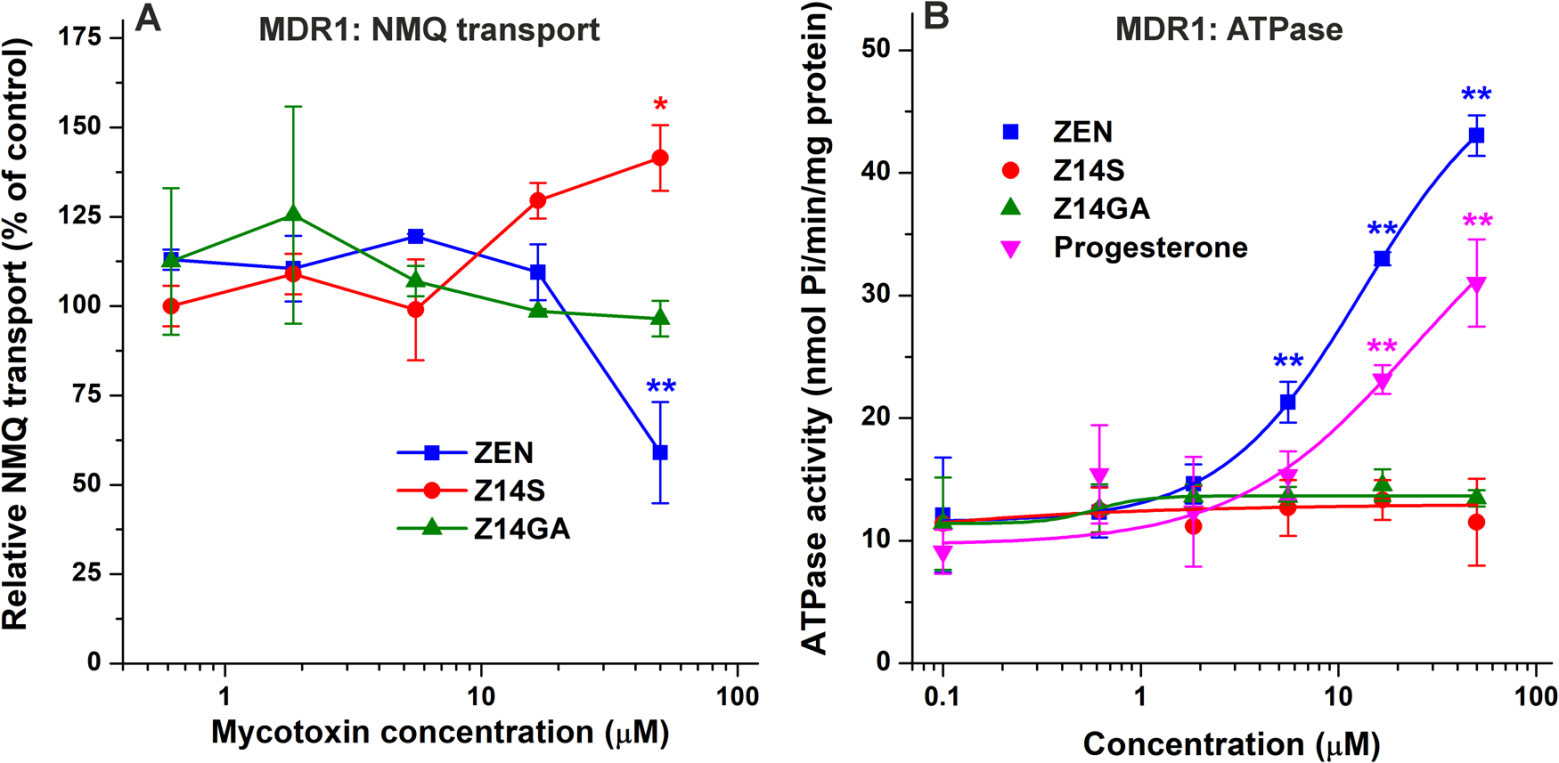
Probing the interactions of ZEN, Z14S, and Z14GA with MDR1. Effects of ZEN and its conjugates on MDR1-mediated NMQ transport (A) and MDR1-dependent vanadium-sensitive ATPase activity (B) in inside-out membrane vesicles. Data represent means ± SD (*n* = 3; *p < 0.05, **p < 0.01).

The BCRP-driven vesicular uptake (Sf9 membrane) of LY was considerably inhibited by ZEN and Z14S, causing more than 90% inhibition at 50 μM concentrations, while Z14GA showed only weak inhibitory action (Figure 5A). The positive control inhibitor Ko143 (1 μM) induced a 95% decrease in LY transport (data not shown). IC_50_ values of ZEN and Z14S were 2.6 and 4.4 μM, respectively. Interestingly, despite its strong inhibitory effect on LY transport, ZEN had no relevant impact on the ATPase activity of BCRP (Figure 5B). We detected only a slight inhibition of basal ATPase activity in the presence of 50 μM ZEN. Z14GA did not affect the BCRP-dependent ATPase activity. Z14S caused a moderate increase even at 2 μM concentration; nevertheless, its impact was lower compared to the reference substrate quercetin (Figure 5B).

**Figure 5:**
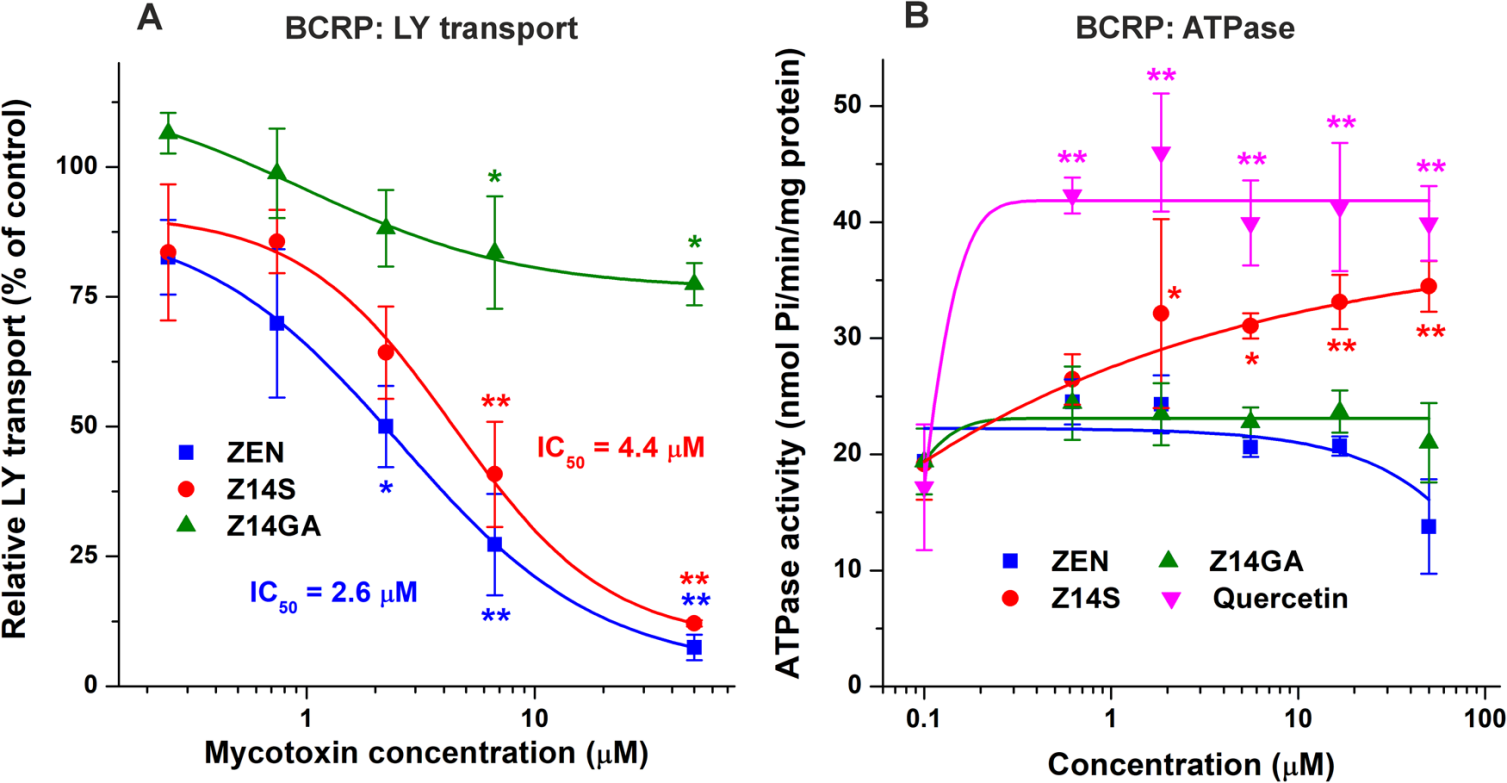
Probing the interactions of ZEN, Z14S, and Z14GA with BCRP. Effects of ZEN and its conjugates on BCRP-mediated LY transport (A) and BCRP-dependent vanadium-sensitive ATPase activity (B) in inside-out membrane vesicles. Data represent means ± SD (*n* = 3; *p < 0.05, **p < 0.01).

The impacts of mycotoxins were also examined on the MRP2-dependent transport of CDCF in the Sf9 vesicular transport assay. Unlike the previous ABC transporters (MDR1 and BCRP), only ZEN conjugates decreased MRP2 activity (Figure 6). Z14S showed a weak inhibitory effect, while Z14GA was a moderate inhibitor (IC_50_ > 10 μM).

**Figure 6:**
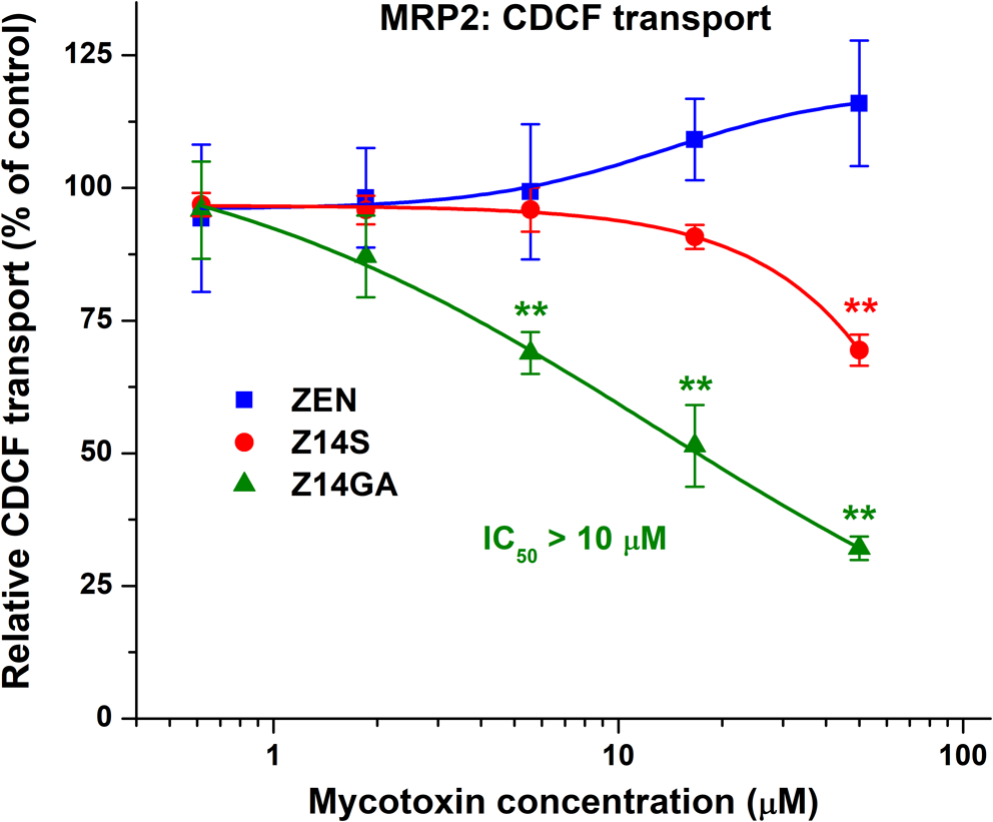
Probing the interactions of ZEN, Z14S, and Z14GA with MRP2. Effects of ZEN and its conjugates on the MRP2-mediated CDCF transport in reverse membrane vesicles. Data represent means ± SD (*n* = 3; **p < 0.01).

The main human multidrug ABC transporters present in important tissue barriers are MDR1 and BCRP. In addition to these, MRP2 is also present, for example, at the canalicular surface of hepatocytes and in the placenta. Our results raise the possibility of the MDR1-mediated transport of ZEN (Figure 4). Nevertheless, earlier transepithelial transport studies suggest no or only minor role of MDR1 in the transport of ZEN.^28,29,31^ Z14S and Z14GA did not show an interaction with MDR1 (Figure 4). Both earlier^29^ and current (Figure 5A) studies demonstrated the inhibitory effects of ZEN on BCRP. Based on our data, ZEN does not appear to be a substrate of BCRP (Figure 5B); however, previous reports suggest the BCRP-mediated transport of the mycotoxin, which can limit the tissue levels and the transplacental transport of ZEN.^29,31^ Thus, BCRP may be able to handle ZEN itself, but the sulfate conjugate of the mycotoxin seems to be a better substrate (Figure 5B). Z14GA showed no or only weak inhibitory actions on MDR1 and BCRP, and it is likely not transported by these carriers (Figures 4 and 5). In contrast, the MRP2-mediated transport of CDCF was inhibited by Z14GA (Figure 6). Since MRP2 has low ATPase activity,^43,47^ we did not perform the ATPase assay with ZEN and its metabolites. Nevertheless, MRP2 commonly transports bulky glucuronic acid conjugates,^22,27^ suggesting that Z14GA may be the substrate of this transporter. Furthermore, our results are also in agreement with the previous general observations that MDR1 has relatively more hydrophobic substrates, BCRP has a preference toward sulfate derivatives, whereas MRP2 is mainly involved in the transport of glucuronic acid conjugates.^22,27^ Taken together, these main multidrug resistance drug transporters may be able to enhance the excretion of ZEN, Z14S, and/or Z14GA and likely take part in the placental defense of the fetus from ZEN-induced toxic impacts.^31^

## 4 Conclusions

The interactions of Z14S and Z14GA were examined with CYP enzymes, OATPs, and ABC transporters. Z14S did not inhibit CYP1A2, while it was a weak inhibitor of CYP2C9 and CYP3A4. The activity of these three CYP enzymes was not affected by Z14GA. Z14S showed strong inhibitory effects on OATPs, with nanomolar (OATP1B1 and OATP2B1) or low micromolar (OATP1A2 and OATP1B3) IC_50_ values. However, Z14GA exerted relatively strong inhibitory effect only on OATP2B1. Among the ABC drug transporters examined, MDR1 showed its most relevant interactions with ZEN, while BCRP with Z14S, and MRP2 with Z14GA. These observations suggest that Z14S and Z14GA have no relevant inhibitory effects on CYP enzymes, while the high exposure to ZEN may affect the OATP-mediated transport of certain compounds. Furthermore, MDR1, BCRP, and MRP2 may be involved in the efflux of ZEN, Z14S, and Z14GA, respectively. As limitations of our study, we have to mention the following two points: (1) OATP-mediated uptake of Z14S and Z14GA was not investigated in the current study, and the possible transport of the mycotoxins by MDR1 and BCRP was examined using an indirect assay (ATPase activity, based on Pi release during the ATP-dependent transport processes). (2) The inhibitory effects on CYPs,^48,49^ OATPs,^50,51^ and ABC transporters^52–54^ can show substrate dependence, and we applied one substrate in each individual assay.

## Supplementary Material



## Notes

Uptake of SR101 or pyranine into A431 cells overexpressing OATP1A2, OATP1B1, OATP1B3, or OATP2B1 transporters and their mock control cells (PDF)
