# Supplementary material for: In Vitro Evaluation of the Potential Interactions of Zearalenone-14-sulfate and Zearalenone-14-glucuronide with Human Cytochrome P450 Enzymes, Organic Anion Transporting Polypeptides, and ATP-Binding Cassette Multidrug Transporters
Source: ACS Omega. 2025 Jul 16;10(29):32466–75. doi: 10.1021/acsomega.5c05217 (PMC12311865; doi:10.1021/acsomega.5c05217)
Supplement: Supplementary file 1 [file ao5c05217_si_001.pdf]

***In vitro* evaluation of the potential interactions of zearalenone-14-sulfate and zearalenone-14-glucuronide with human cytochrome P450 enzymes, organic anion transporting polypeptides and ATP-binding cassette multidrug transporters**

SUPPLEMENTARY MATERIALS

Ágnes Telbisz [1], Hana Kaci [2,3], Éva Bakos [2], Zoltán Nagymihály [4,5], Eszter B. Both [4], Nándor Lambert [4,6], Csilla Özvegy-Laczka [2], Miklós Poór [5,7,*]

[1] Gene Regulation Research Group, Institute of Molecular Life Sciences, Research Centre for Natural Sciences, HUN-REN, Budapest, Hungary

[2] Drug Resistance Research Group, Institute of Molecular Life Sciences, Research Centre for Natural Sciences, HUN-REN, Budapest, Hungary

[3] Doctoral School of Biology, Institute of Biology, Eötvös Loránd University, Budapest, Hungary

[4] Soft Flow Ltd., Pellérdi út 91/B, H-7634 Pécs, Hungary

[5] Molecular Medicine Research Group, János Szentágothai Research Centre, University of Pécs, Ifjúság útja 20, H-7624 Pécs, Hungary

[6] National Laboratory on Human Reproduction, University of Pécs, Ifjúság útja 20, H-7624 Pécs, Hungary

[7] Department of Laboratory Medicine, Medical School, University of Pécs, Ifjúság útja 13, H-7624 Pécs, Hungary

*Corresponding author: Miklós Poór, PharmD, PhD
Department of Laboratory Medicine,
Medical School, University of Pécs
Ifjúság útja 13, H-7624 Pécs, Hungary
Phone: +36-72-501-500 ext: 29250
E-mail: poor.miklos@pte.hu

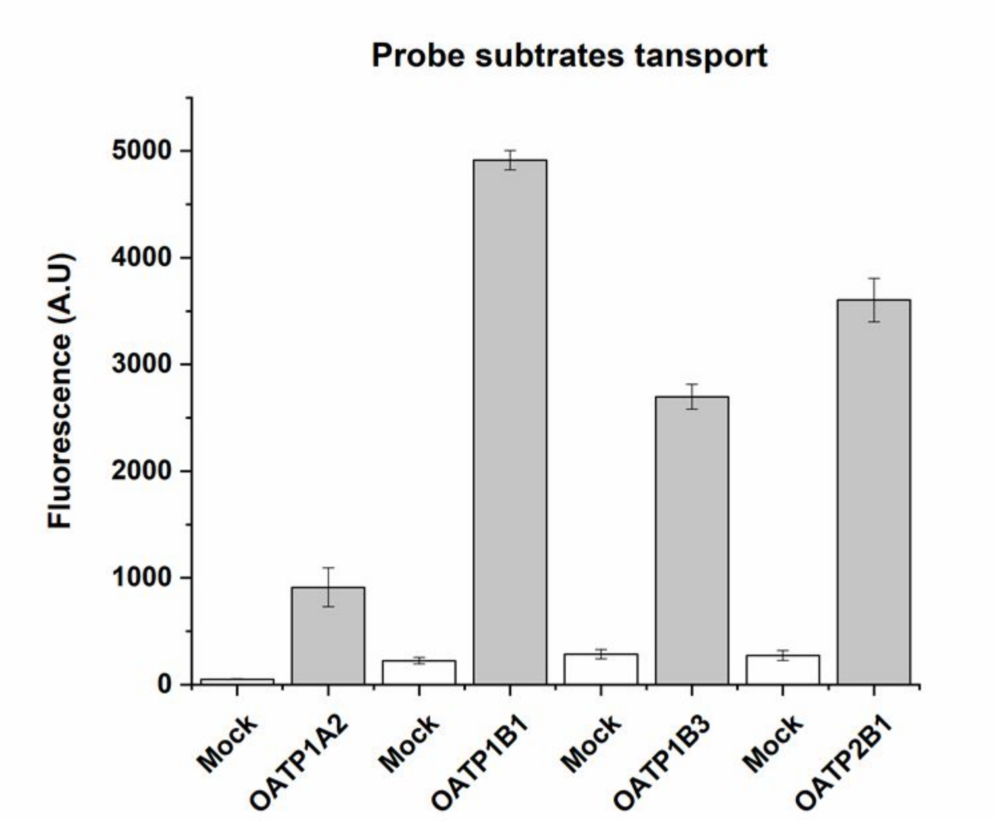

Fig. S1: The uptake of SR101 or pyranine into A431 cells overexpressing OATP1A2, OATP1B1, OATP1B3, or OATP2B1 transporters, and their mock control cells. A431-OATP1A2, -OATP1B1, -OATP1B3 or -OATP2B1 cells, or their mock-transfected controls were incubated with fluorescent substrates (SR101 (0.5 µM, OATP1A2) or pyranine (10 µM for OATP1B1, or 20 µM for OATP1B3 and OATP2B1)) at 37 ºC in uptake buffer (pH 5.5) for 10 min (OATP1A2), 15 min (OATP1B1 and OATP2B1), or 30 min (OATP1B3) incubation. The transport of probe substrates was measured using an Enspire plate reader (PerkinElmer, Waltham, MA, US) at ex/em wavelengths of 460/510 nm for pyranine or 586/605 nm for SR101. Data are presented as means ± SD (n = 3).
